# Role of Substrate Type in the Process of Polyelectrolyte Multilayer Formation

**DOI:** 10.3390/polym14132566

**Published:** 2022-06-24

**Authors:** Mia Mesić, Tin Klačić, Anže Abram, Klemen Bohinc, Davor Kovačević

**Affiliations:** 1 Department of Chemistry, Division of Physical Chemistry, Faculty of Science, University of Zagreb, 10000 Zagreb, Croatia; mmesic@chem.pmf.hr (M.M.); tklacic@chem.pmf.hr (T.K.); 2 Department for Nanostructured Materials, Jožef Stefan Institute, 1000 Ljubljana, Slovenia; anze.abram@ijs.si; 3 Faculty of Health Sciences, University of Ljubljana, 1000 Ljubljana, Slovenia; klemen.bohinc@zf.uni-lj.si

**Keywords:** polyelectrolyte multilayers, silicon, titanium, AFM, tensiometry, ellipsometry, streaming potential

## Abstract

Polyelectrolyte multilayers are coatings formed by the alternate deposition of polycations and polyanions on a charged surface. In this study we examined how the type of substrate affects a multilayer prepared from poly(allylamine hydrochloride) and poly(acrylic acid). Silicon and titanium wafers were used as substrates. Their properties were systematically studied using ellipsometry, tensiometry, atomic force microscopy and streaming potential measurements. Multilayers were built up at pH = 7 with tetramethylammonium chloride as the background salt. The growth of films was monitored by ellipsometry, while the morphology and surface roughness were determined by atomic force microscopy. It was found that the thickness of multilayers containing 10 layers on silicon is 10 nm, whereas the thickness of the same film on titanium is three times higher. It was shown that multilayers formed on silicon display a grain-like structure, which was not the case for a film formed on titanium. Such morphological properties are also reflected in the surface roughness. Finally, it was shown that, in addition to the electrostatic interactions, the hydrophobicity of the substrate also plays an important role in the polyelectrolyte multilayer formation process and influences its thickness and properties.

## 1. Introduction

Polyelectrolytes are macromolecules that contain ionic or ionizable functional groups. The physico-chemical properties of polyelectrolytes depend strongly on the interactions between charged monomers. Moreover, these properties are also influenced by the condensation of counterions on charged functional groups. In addition to the ionic condensation, high-density polyelectrolytes are also known for the pronounced tendency to react with oppositely charged polyelectrolytes and/or with charged surfaces, such as metal oxide surfaces [1]. These processes lead to the formation of polyelectrolyte complexes [2,3] and polyelectrolyte multilayers (PEMs) [4,5], respectively. The outcome of interpolyelectrolyte neutralization at surfaces can be often predicted on the basis of complexation experiments and vice versa [6].

Polyelectrolyte multilayers are most often prepared by the layer-by-layer (LbL) method proposed by Decher [7]. The substrate is usually immersed in the polyelectrolyte solution of the desired concentration and ionic strength followed by rinsing with water or an appropriate solvent and, if necessary, by drying with an inert gas. The procedure is repeated several times until the desired number of layers is obtained. The surface of the substrate is electrically charged, so the first polyelectrolyte layer always bears the opposite charge compared to the surface (i.e., overcharging takes place), which enables the adsorption of the next polyelectrolyte of opposite charge. The multilayer can be prepared on a plate or on a particle. For the formation of a multilayer, electrostatic interactions often play the crucial role. However, non-electrostatic forces such as hydrogen bonds, as well as van der Waals and hydrophobic interactions, could also influence multilayer build-up. In addition to the dipping method, other methods for the preparation of PEMs are also known, such as spin coating, spraying or flowing the solution onto the substrate surface [8,9,10].

The properties of multilayers, in addition to the type of polyelectrolyte used, are mostly influenced by the pH value, ionic strength, type of background salt, temperature and type of solvent [11,12,13,14]. Many researchers have studied the influence of pH on the growth, stability and properties of a PEM formed by the layer-by-layer method. Schönhoff and Bieker [15], as well as Shiratori and Rubner [16,17], studied how pH affects a polyelectrolyte multilayer made of weak polyelectrolytes poly(allylamine hydrochloride), PAH, and poly(acrylic acid), PAA. It has been observed that the change in pH influences the growth mechanics, thickness, surface morphology and surface wettability. An atomic force microscopy study [18] showed, on the example of PEMs composed of poly-L-lysine and heparin, that the surface roughness increases with increasing pH value. In addition to pH value, it was shown that the ionic strength and type of background salt are also important for the stability, permeability, structure, function, growth and electrostatic interactions of multilayers [16]. High salt concentrations shrink and create softer multilayers, while thickness and roughness increase with increasing salt concentration [17]. The formation and stability of polyelectrolyte multilayers are generally affected by the competition of interactions between charged groups of polyanions and polycations and their interactions with small counterions [19].

A variety of materials could be used as substrates for PEM formation. Typical examples are glass, quartz, silicon wafers, mica and gold-coated supports [20]. Barrantes et al. [18] investigated how the surface properties of the substrate (Si and Au) affect the multilayer formation and properties. It was shown that substrate type, as well as substrate refractive index, roughness and surface charge influence the build-up of poly-L-lysine/heparin multilayers. Buron et al. [21] have used several chemically modified types of silica (bare silica, aminated silica and two hydrophobically modified types of silica) as the substrates for the formation of polyelectrolyte multilayers formed from poly(trimethylammonium ethyl methacrylate chloride) (MADQUAT) and poly(acrylic acid). On all examined substrates, the growth was exponential in the range of the first five bilayers and there was a very strong dependence of the growth on the functionality of the substrate.

Several studies have also shown that substrate geometry could affect PEM build-up and properties. For practical reasons, flat substrates are mostly used nowadays for the preparation of multilayers. Porous materials are also sometimes used. However, it was found that more cycles of immersion in solutions are required for the successful coverage of the pores [22]. Spherical colloidal particles are also often used as templates for obtaining polyelectrolyte multilayers and/or hollow capsules which have wide application in biomedicine, mainly for drug delivery purposes [23,24,25].

As stated above, the application of polyelectrolyte multilayers in various fields is very promising. In the literature, there are already many examples of their application, e.g., in the field of membranes [26]. Moreover, there are many studies dealing with surface modifications on the basis of polyelectrolyte multilayers with the aim to obtain surfaces with antibacterial [27,28], sensory [29] or biocompatible properties [30].

The main aim of this study was to compare the influence of two metal substrates (silicon and titanium) on the polyelectrolyte multilayer film build-up on the example of a PEM prepared from weak polyelectrolytes, poly(allylamine hydrochloride) and poly(acrylic acid). For that purpose, systematic characterization of both the substrate and formed multilayer is needed. Therefore, special emphasis was given to the characterization of silicon and titanium substrates in terms of their properties such as roughness, charge and hydrophobicity. The same methods were applied for the characterization of PEMs. Such investigations are needed in order to examine the role of the substrate in the process of polyelectrolyte multilayer build-up, which could lead to the formation of PEMs with tunable thickness and properties.

## 2. Materials and Methods

### 2.1. Materials

Silicon and titanium wafers were used as substrates: single-side polished monocrystalline silicon discs, p-type; orientation, 100; doping, B; diameter, 150 mm; thickness, (675 ± 25) μm (Siltronic AG, München, Germany) and single-side polished polycrystalline titanium wafers, purity > 99.9%; dimensions, 10 × 10 × 0.5 mm^3^ (MTI Corporation, Richmond, CA, USA). Silicon discs were cut into strips with dimensions of 10 mm × 10 mm, cleaned in *piranha* solution and rinsed with deionized water. Titanium wafers were cleaned with absolute ethanol in an ultrasonic bath for 10 min and then rinsed with deionized water. All substrates were dried with inert argon.

For tensiometry measurements, the following liquids were used: glycerol (*w* ≥ 99%), dimethyl sulfoxide (*w* ≥ 99.8%), toluene (*w* ≥ 99.8%), formamide (*w* ≥ 99.5%), *N*,*N*-dimethylformamide (*w* ≥ 99.8%) (all obtained from Sigma Aldrich, St. Louis, MO, USA) and deionized water.

The polyelectrolytes we used were poly(allylamine hydrochloride), PAH, *M_w_* ≈ 17,500 g mol^−1^, degree of polymer functionalization *f* = 0.882, as a polycation and poly(acrylic acid), PAA, *M_w_* ≈ 1,033,000 g mol^−1^, *M_n_* ≈ 239,300 g mol^−1^, *f* = 0.969, as a polyanion. Both polyelectrolytes were obtained from Sigma Aldrich, St. Louis, MO, USA.

Polyelectrolytes (*c* = 0.01 mol dm^–3^), salt tetramethylammonium chloride, Me_4_NCl, *w* ≥ 97% (Sigma Aldrich, St. Louis, MO, USA, purified by recrystallization), and buffer MOPS (3-(N-morpholino)propane-1-sulfonic acid) (Sigma Aldrich, St. Louis, MO, USA) were dissolved in deionized water. pH values of prepared polyelectrolyte solutions were adjusted to pH = 7.0 ± 0.1 by the addition of NaOH aqueous solution (Merck, Darmstadt, Germany). This pH value was chosen to achieve the optimal degree of ionization of both weakly charged polyelectrolytes used for the multilayer assembly [31]; at this pH, both polyelectrolytes are charged between 70 and 85% of full dissociation.

Polyelectrolyte multilayers were assembled in the following way: Since both examined surfaces at pH = 7 are negatively charged, the cleaned substrate was first immersed in PAH solution for 5 min. During the adsorption process, solution was stirred with a magnetic stirrer (v ≈ 500 rpm) at room temperature. After that, it was rinsed with deionized water three times for one minute to wash away loosely bound polyelectrolyte chains. Before the adsorption of the next layer, the substrate was dried with argon. Then, the substrate was immersed in PAA solution in the same manner as stated above. The whole process was then repeated until the desired number of layers was obtained.

### 2.2. Methods

#### 2.2.1. Ellipsometry

The measurements were performed using the *Ellipsometer L116B-USB* (Gaertner Scientific Corporation, Skokie, IL, USA) instrument. Experiments were carried out at (24 ± 2) °C and at a relative humidity of 30 to 50% by monochromatic laser beam (*λ* = 632.8 nm) incident on the sample surface at an angle of 70°. For calculation of thickness, the commercial *Gaertner Ellipsometric Measurement Program* (Version 8.071) was used. In the software, a three-box model was assumed with air as a continuum (*n* = 1.00) [32], oxide layer (*n* = 1.457 for SiO_x_ [33] and *n* = 2.130 for TiO_x_ [34]) or PEM (*n* = 1.55 [35]) as a film and silicon or titanium wafer as a substrate. While for the oxide layer determination pure silicon (*n* = 3.873 and *k* = 0.016 [36]) or pure titanium (*n* = 2.704 and *k* = 3.765 [37]) was taken as a substrate, for PEM thickness determination the metal/metal oxide substrate was treated as a one-phase system and, before film thickness measurements, its average refractive index was determined by ellipsometric measurements on ten different positions on each used plate.

#### 2.2.2. Atomic Force Microscopy

Atomic force microscopy (AFM) measurements were performed under ambient air conditions at (25 ± 2) °C and relative humidity between 20 and 55% on a *Multimode 8 AFM* apparatus (Bruker, Billerica, MA, USA). The surface topography of metal/metal oxide substrates was visualized in contact mode, whereas tapping mode was used to image the surface of polyelectrolyte multilayers. For the measurements in contact mode, *ScanAssyst Air* probes (Bruker, Billerica, MA, USA) with a nominal resonance frequency of 70 kHz, a nominal spring constant of 0.4 N m^−1^ and a tip with a nominal radius of curvature of 2 nm were used. For imaging in tapping mode, the NCHV-A probes (Bruker, Billerica, MA, USA) of a resonance frequency of approximately 320 kHz, a nominal spring constant of 42 N m^−1^ and a tip with a nominal radius of curvature of 8 nm were used. All measurements were done on a 5 μm × 5 μm area with a scanning rate of 0.5–1.0 Hz and image resolution of 515 × 512 pixels. After the data were processed in *NanoScope Scan 9.7*, AFM images were corrected for tilt and bow using a second-order flattening and were analyzed in *NanoScope Analyses 2.0* software to determine the root mean square (RMS) roughness of surface materials. The AFM roughness parameters and appropriate standard errors reported here were calculated from all the measurements, which included five local areas on the sample surfaces. For measuring the film thickness by AFM, a step-edge boundary between the PAH/PAA multilayer and substrate surface was made. In the case of Si samples, this was achieved by gently scribing the surface with stainless steel microscope tweezers, while in the case of Ti samples the boundary was created by covering one part of the surface with *Parafilm M* (Bemis, Sheboygan Falls, WI, USA) prior to multilayer formation. Then, the measurements were made by scanning the AFM tip across each step-edge boundary at a right angle. Finally, the film thickness was taken from the height profile by placing the cursors on the multilayer and substrate area and taking the height difference at each position. A minimum of 10 such measurements were made for each thickness determination, with the average film thickness reported. The error for the film thickness is reported as the standard error of the mean.

#### 2.2.3. Contact Angle Measurements

Measurements of contact angles were performed using an *Attension Theta T200-Basic Plus* (Biolin Scientific, Espoo, Finland) tensiometer with six different test liquids on previously cleaned substrates. The test liquids we chose were: deionized water, glycerol, toluene, *N*,*N*-dimethylformamide, dimethyl sulfoxide and formamide. The purity of the liquids is stated in Section 2.1. Before the measurements were completed, it was necessary to calibrate the instrument with a tungsten carbide ball. Tensiometer calibration, experiments and data analysis were performed in the computer program *OneAttension* (version 3.2). Measurements of the contact angle were performed using the sessile drop method at the room temperature of 25 °C, atmospheric pressure and relative humidity of 50 to 55%. The drop of the test liquid was approximately 5 μL. The recording lasted 10 s at a frequency of 331 photos per second. The photographs were stored on a computer and the droplet contour on the substrate was processed by the Young–Laplace equation on a sample of 100 photographs between the third and sixth seconds of shooting. The contact angle is an average value of five measurements.

#### 2.2.4. Streaming Potential Measurements

Streaming potential measurements were performed using a *SurPASS electrokinetic analyzer* (Anton Paar, Graz, Austria) using tetramethylammonium hydroxide solution (Sigma Aldrich, St. Louis, MO, USA) for adjusting pH, with tetramethylammonium chloride (Me_4_NCl, *c* = 0.01 mol dm^−3^) as the background electrolyte. At room temperature, the solution was forced to flow through a capillary and the electrical potential was produced between the ends of the capillary. The obtained electrical difference is denoted by the streaming potential. The zeta potential was calculated from the streaming potential using the Helmholtz–Smoluchowski equation.

## 3. Results and Discussion

### 3.1. Substrate Characterization

Both examined metal surfaces were systematically studied by means of various techniques. Oxide layer thickness was examined ellipsometrically, AFM and contact angle measurements were applied for the evaluation of surface roughness and wettability, while streaming potential measurements were used for the zeta potential determination.

#### 3.1.1. Oxide Layer Thickness

A parameter that significantly influences the behaviour of a certain metal oxide surface is the thickness of the oxide layer. The oxide layer is spontaneously formed by oxidation with the oxygen from the air. The oxide layer was examined ellipsometrically for both studied substrates (silicon and titanium) and the thickness was determined (Table 1).

From the results presented in Table 1, it could be concluded that both oxide layers are a few nanometers thick. The thickness values obtained in our study are in accordance with the literature values [38,39]. Very low experimental errors (only 0.02 nm) show the high reproducibility of the ellipsometrically obtained results, but also demonstrate the uniformity of the oxide layers at the metal surface.

#### 3.1.2. Surface Morphology and Roughness

**Figure 1:**
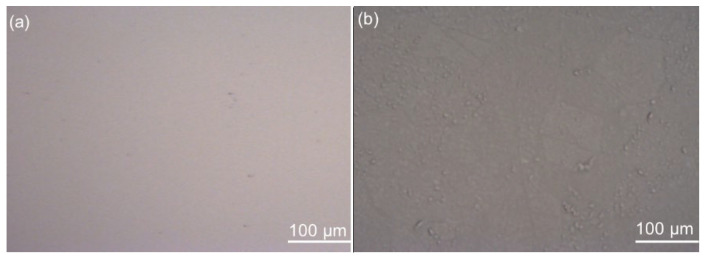
Images (500 μm × 400 μm) of the surface of (**a**) Si substrate and (**b**) Ti substrate obtained by digital optical microscopy.

Atomic force microscopy was used to determine the morphology of the substrate surface. Before imaging the surface with AFM, images on a larger scale were made using a digital optical microscope (Figure 1). From the images obtained by the digital optical microscope, it is clear that the surface of silicon is very smooth with only a few impurities or defects on the surface. Although the titanium is somewhat rougher than silicon, and despite minor defects and impurities on both surfaces, the general conclusion is that the surfaces of both metal samples, at least on a micrometer scale, are very smooth.

**Figure 2:**
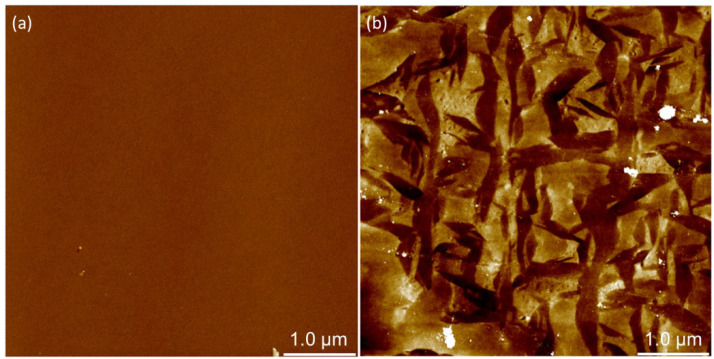
AFM images (5 μm × 5 μm) of (**a**) Si substrate and (**b**) Ti substrate surfaces. Higher areas are represented by lighter colours. Maximal value of the *z*-axis is 5 nm.

A comparison of AFM images of the substrate surfaces (Figure 2) shows that the silicon surface is almost perfectly smooth with only a few impurities or defects. Unlike the almost perfectly smooth silicon surface, the titanium surface is somewhat rougher and is characterized by many terraces and irregularities. In order to quantify the roughness, AFM images of the substrate surface were analyzed and values for RMS roughness were obtained as *R*_q_ = (0.28 ± 0.05) nm for the silicon and *R*_q_ = (1.57 ± 0.19) nm for the titanium surface. What is immediately noticeable from the obtained values of the roughness parameters (roughness below 2 nm) is that both studied surfaces belong to the category of extremely smooth surfaces. Furthermore, a comparison of *R*_q_ values shows that the silicon surface is smoother than the titanium surface. Although there are small differences in the surface roughness of the investigated samples, it was concluded that the examined Si and Ti plates are sufficiently smooth to be used as metal substrates for the preparation of polyelectrolyte multilayers.

#### 3.1.3. Zeta Potential

In the case of metal oxide wafers, streaming potential measurements are often performed with the aim to determine the isoelectric point [40]. From the isoelectric point value, the predictions of the sign and value of the zeta potential (and consequently the surface charge) at various pH values could be obtained. Therefore, we performed streaming potential measurements of silica and titania flat surfaces to obtain the isoelectric point (pH_iep_) of both studied surfaces. For the silica surface, pH_iep_ was determined to be 3.5 ± 0.2, and for the titania surface, pH_iep_ = 4.1 ± 0.2. These results are in accordance with the values from the literature claiming that both for particles and flat surfaces the isoelectric point of titania is in principle higher than the isoelectric point of silica [41]. For example, Roessler and coworkers [42] showed that titanium sputtered on glass has an IEP of 4.4 and hence is negatively charged at physiological pH. On the other hand, Wasilewska and coworkers applied streaming potential measurements for the determination of the zeta potential of the oxidized Si/SiO_2_ wafers and showed that the substrate was negative for all pHs studied, varying between −16 and −49 mV at pH 3.5 and 7.4, respectively (for *I_c_* = 10^−2^ mol dm^−3^ NaCl) [43]. Additionally, Brkljača et al. showed that for the quartz/aqueous electrolyte solution interface pH_iep_ was found to be below 3 [44].

#### 3.1.4. Contact Angles

Tensiometric measurements were performed to determine the proportion of polar and non-polar interactions between substrates and polyelectrolyte molecules. Contact angles were determined for both studied substrates using six different test liquids on previously cleaned substrates. In order to obtain the percentage of polar and non-polar interactions in a particular substrate, we used the Owens–Wendt model, whose linearized form is shown by the following equation:

(1) $$\frac{\sigma_{L}\;\left(1+\cos\theta \right)}{2{\left(\sigma_{L}^{d}\right)}^{\frac{1}{2}}}={\left(\sigma_{S}^{p}\right)}^{\frac{1}{2}}\;{\left(\sigma_{L}^{p}/\sigma_{L}^{d}\right)}^{\frac{1}{2}}+{\left(\sigma_{S}^{d}\right)}^{\frac{1}{2}}$$

where *θ* is the contact angle between the solid surface and liquid, *σ*_L_ represents the surface tension of a liquid, $\sigma_{L}^{p}$ is a polar component of the surface free energy of a liquid and $\sigma_{L}^{d}$ represents the dispersion component of the surface free energy of a liquid. These values can be found in the literature (Table 2). The interpretation of the results leads to the values of $\sigma_{s}^{p}$, i.e., the polar component of the surface free energy at the solid/gas interface and to the values of $\sigma_{s}^{d}$, i.e., the dispersion component of the surface free energy at the solid/gas interface, for which the ratio between polar and non-polar interactions could be determined.

**Figure 3:**
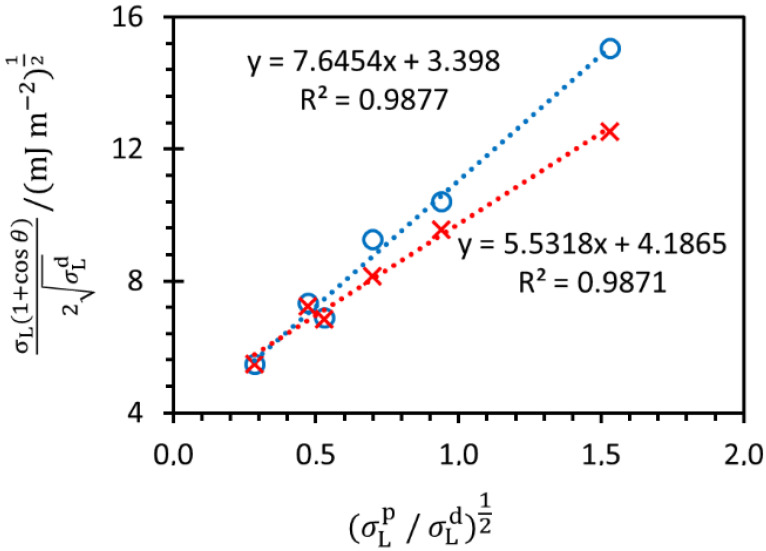
Results for silica (**O**) and titanium (**X**) contact angle measurements with six different test fluids interpreted by Owens–Wendt model (Equation (1)).

In Figure 3, the results determined by applying the Owens–Wendt model on the experimentally obtained values of contact angles with six different test liquids are presented. For all tensiometric measurements we used two samples of each metal substrate and we performed five measurements on each sample, so the following results are actually the average of ten measurements on each metal substrate.

The values of $\sigma_{s}^{p}$ and $\sigma_{s}^{d}$ for each sample were calculated from the slope and intercept which are presented in Figure 3. From these values, the total surface free energies of the silicon substrate (*σ*_s_ = 70.0 mJ m^−2^) and titanium substrate (*σ*_s_ = 48.2 mJ m^−2^) were obtained. Silicon substrate has a higher surface free energy than titanium substrate, which means that the silicon surface is generally more hydrophilic. Furthermore, the percentage of polar and non-polar interactions for each substrate was calculated on the basis of the Owens–Wendt model and the percentage of polar interactions was found to be 84% in the case of the silicon surface and 64% in the case of the titanium surface. On the basis of the obtained results, it could be concluded that silicon substrate in comparison to titanium substrate creates a higher amount of stronger polar interactions (e.g., hydrogen bond) with hydrophilic polymers such as PAH and PAA.

### 3.2. Polyelectrolyte Multilayer Formation and Characterization

#### 3.2.1. Multilayer Thickness Obtained by Ellipsometry

**Figure 4:**
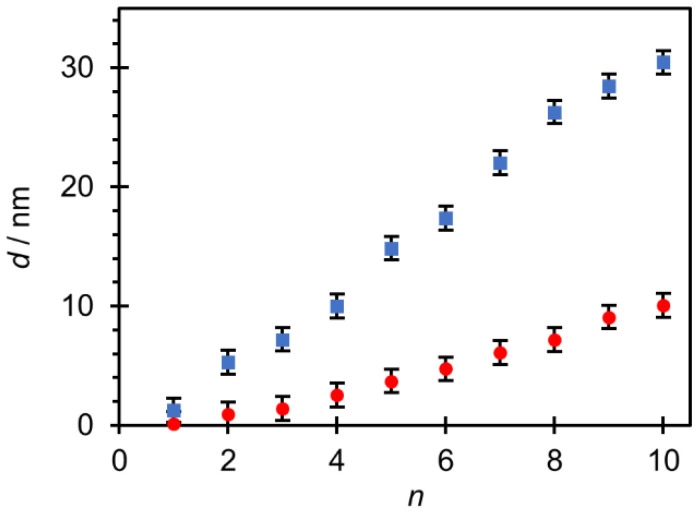
Thickness of PAH/PAA multilayers on Si (●) and Ti (■) substrates determined by ellipsometry presented as a function of the number of deposited polyelectrolyte layers.

Polyelectrolyte multilayers were prepared on both studied surfaces by alternately immersing the substrate in polyelectrolyte solutions (PAH and PAA) as described in the *Materials and Methods* section. As few studies [49,50] have demonstrated that short chain polyelectrolytes tend to form rather unstable PEM systems due to the stripping of polymers from the multilayer surface and the formation of soluble polyelectrolyte complexes (PECs), we decided to use PAA of a high molecular weight and PAH of a low molecular weight in this study. Since at pH = 7 both surfaces were negatively charged, the first polyelectrolyte added was polycation PAH, then PAA and so on. The resulting film had a total of ten layers, and the thickness of the film was determined using ellipsometry (Figure 4). The PAH/PAA multilayer prepared on silicon is thinner and its thickness is only 10.1 nm, while on titanium it is three times thicker and is 30.5 nm. Figure 4 shows that the growth of both films could be defined as linear.

#### 3.2.2. Multilayer Thickness Obtained by Atomic Force Microscopy

**Figure 5:**
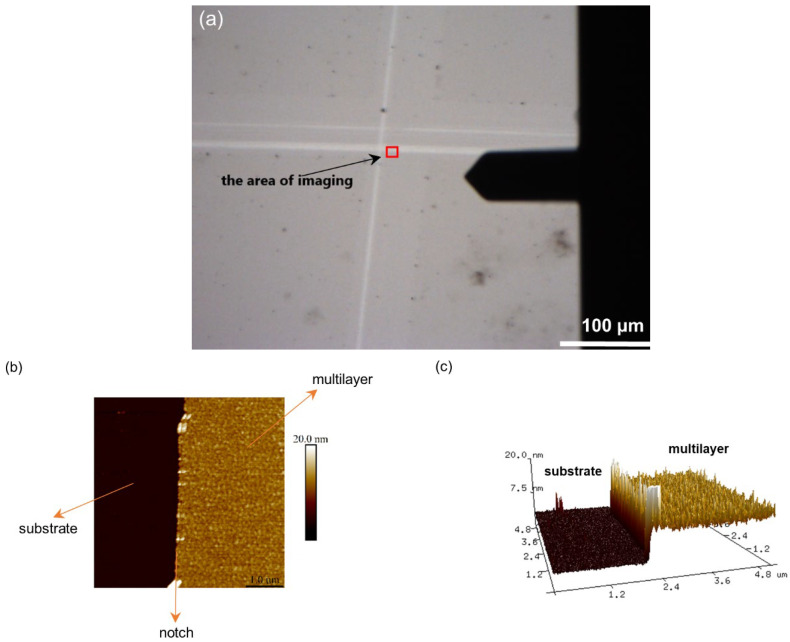
The area where PAH/PAA multilayer was removed from the surface of the Si substrate, recorded by (**a**) digital optical microscope and (**b**,**c**) atomic force microscope. Figure (**b**) presents a 2D AFM image of the surface, and Figure (**c**) a 3D AFM image.

To determine the thickness of the PAH/PAA multilayers on the silicon substrate using AFM, the multilayer was partially removed so that a scratch could be made on the surface with sharp-tipped tweezers. The surface was then imaged in the scratched area with a digital optical microscope and AFM (Figure 5).

**Figure 6:**
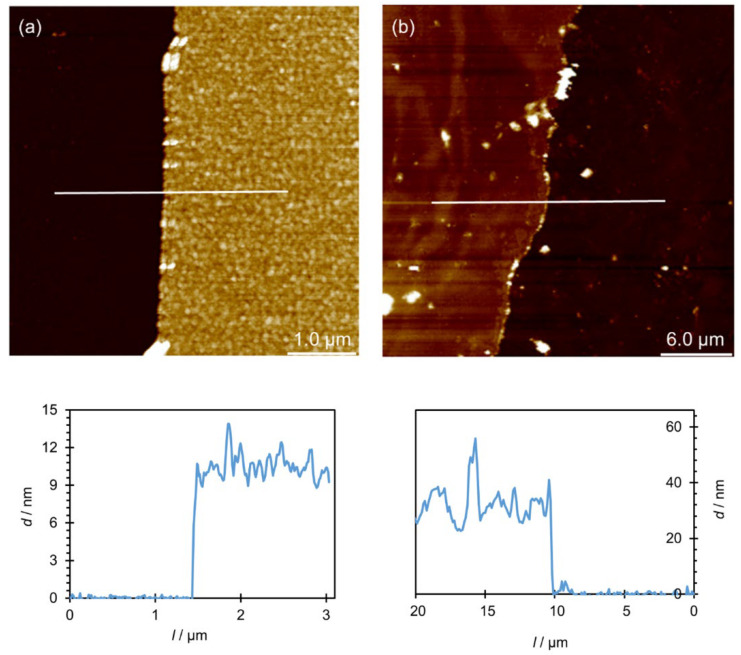
AFM images of step-edge boundary between (PAH/PAA)_5_ multilayer and (**a**) silicon or (**b**) titanium surface. The corresponding height profiles are shown below the images.

In Figure 5a, the white lines represent parts of the surface from which the polyelectrolyte film was removed. If AFM was used for a more detailed observation at the area along the very edge of these lines (Figure 5b), a flat surface of the substrate could be observed, to which the rougher surface of the multilayer was “connected”. Unlike silicon, it was not possible to make scratches on titanium with tweezers because the substrate is too soft and such a procedure would only damage it. Therefore, for that purpose *Parafilm M* was placed on approximately half of the plate to protect that part of the substrate from multilayer coating. Although the applied method in titanium resulted in a less noticeable boundary between the film and the substrate, the thickness on that substrate was also successfully determined. Finally, the thickness of the PAH/PAA multilayer on Si and Ti was determined by pulling the height profiles at ten different locations at the substrate/film boundary (Figure 6). Based on the difference in the average film surface height and the average substrate surface height, the average film thickness value and its standard error were determined. The results are presented in Table 3 and compared with the results obtained by ellipsometry.

From the results presented in Table 3, two conclusions could be made. The first is that, with regard to the type of metal substrate, there is a growing trend in the thickness of the PAH/PAA multilayers obtained by both applied methods when we compare silicon with titanium. While the thickness of a studied multilayer on silicon is only *cca* ten nanometers, the thickness of the same LbL film on titanium is three times higher. The same trend is observed with the standard error of the film thicknesses on silicon and titanium. Such an increase in standard errors suggests that the morphology and surface roughness of the PAH/PAA multilayer are not the same on both metal substrates, which will be discussed in more detail in the next section. The second conclusion is that the thickness of multilayers determined by AFM is slightly lower than the thickness determined by ellipsometry. A similar observation was noted by Salomäki et al. [51] for PDADMAC/PSS multilayers formed on a silicon substrate. It could be assumed that this difference in results is actually a consequence of the methods themselves because AFM is the absolute method and gives a realistic picture of the results, while ellipsometry is based on the model and the average thickness is being calculated. The height profiles obtained by processing AFM images (Figure 6) actually show a realistic image of the multilayer, where it is seen that the multilayer is not flat but that there are larger and smaller bumps on the surface.

#### 3.2.3. Morphology and Roughness of PEM Surface

**Figure 7:**
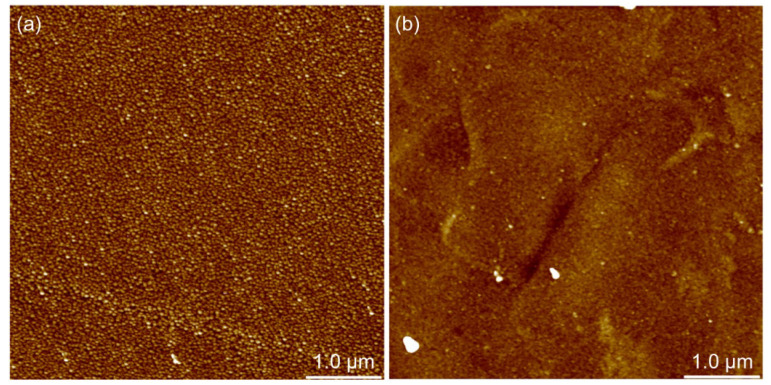
AFM image of 5 μm × 5 μm surface of PAH/PAA multilayer made of 2 bilayers on (**a**) Si substrate and (**b**) Ti substrate. The higher areas are shown in a lighter color; z-scale value is 20 nm.

**Figure 8:**
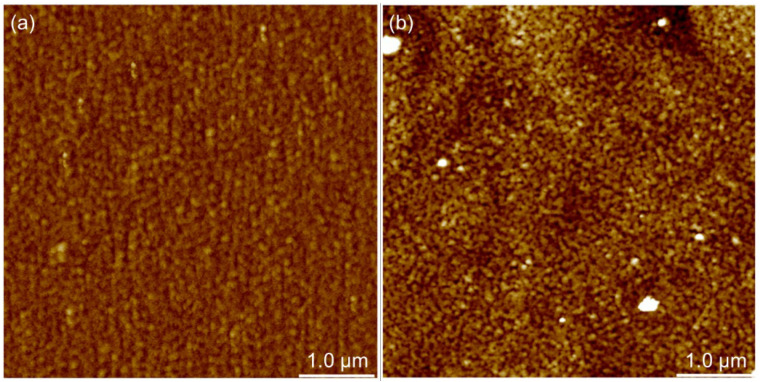
AFM image of 5 μm × 5 μm surface of PAH/PAA multilayer made of 5 bilayers on (**a**) Si substrate and (**b**) Ti substrate. The higher areas are shown in a lighter color; z-scale value is 20 nm.

It has already been noted in the previous chapter that PAH/PAA multilayers prepared on different substrates do not have the same morphological characteristics. In order to investigate the influence of the substrate type on the morphological characteristics of these thin films, PAH/PAA multilayers of a total of four and ten layers on each of the substrates were prepared. In both cases (4 and 10 layers) PAH/PAA film was prepared, and the topography of the film surface on silicon and titanium (Figure 7 and Figure 8) was recorded using the tapping mode of AFM.

From the AFM images of the film containing four layers (Figure 7) it could be concluded that the film formed on the silicon surface has a granular morphology, while in the case of titanium, terraces could be observed. From the height profiles presented in Figure 6, it could be observed that (PAH/PAA)_5_ multilayers prepared on both metal substrates are compact and non-porous, and Figure 8 confirms this by providing an even more detailed insight into the morphology of these films.

**Figure 9:**
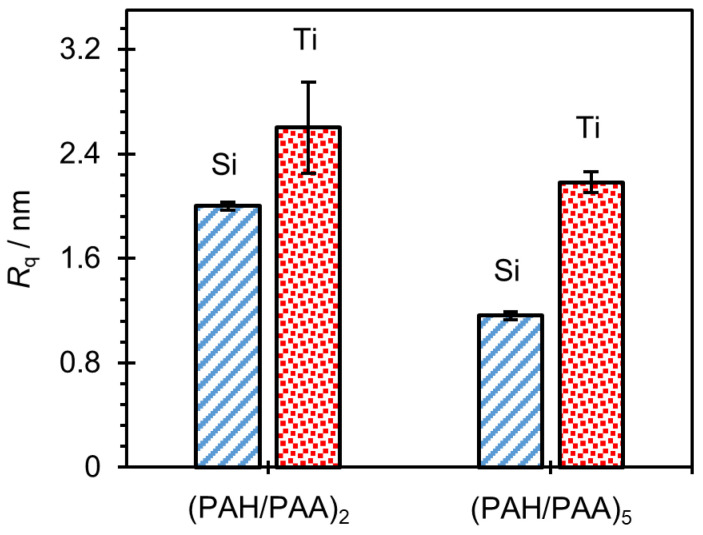
Root mean square surface roughness (*R*_q_) obtained for silicon and titanium surfaces coated with 2 or 5 PAH/PAA bilayers.

If we now compare the topography of the LbL film surface with two and five PAH/PAA bilayers, several conclusions can be drawn. First, the surfaces of (PAH/PAA)_2_ and (PAH/PAA)_5_ multilayers formed on silicon and titanium have similar morphological characteristics. However, in the case of a film with five bilayers, the grain structure of the surface is somewhat more pronounced. Second, the greatest difference in morphology is observed in the multilayer formed on titanium. While a four-layer film on a Ti substrate has a surface with extremely fine grains, a ten-layer film has a “worm-like” surface. Also, the morphological properties of the surface of the prepared films are reflected in the surface roughness of the samples. By additional processing of the AFM images, values of the root mean square (RMS) roughness parameter for (PAH/PAA)_2_ and (PAH/PAA)_5_ multilayers prepared on Si and Ti substrates were obtained (Figure 9).

As depicted in Figure 9, PAH/PAA multilayers prepared on the titanium surface are rougher than those prepared on the silicon surface. In addition to the influence of the substrate type, the morphology and surface roughness of a PAH/PAA multilayer strongly depend on the molecular weight of the polyelectrolytes. The high molecular weight polyelectrolytes could facilitate the generation of larger islet-like structures on the surface as compared to the low molecular weight polyelectrolytes. As a result, PEMs made of long chain polyelectrolytes are rougher than multilayers made of short chain polyelectrolytes. For example, Yu et al. [52] reported that the surface roughness of the PAH/PAA multilayer increased from 3.7 nm to 141.3 nm with the increase of the molecular weight of PAA from 15,000 to 225,000 g/mol. The influence of molecular weight also reflects on the film thickness. In general, for linear growth behavior, as in our case (Figure 4), the film thickness increases with the increase of chain length of polyelectrolytes because PEM contains more coiled polyelectrolyte chains [49,50].

If we summarize all the obtained results, it is obvious that the thickness and the surface roughness of (PAH/PAA)_5_ multilayers are higher for the Ti surface compared to the Si surface. In the literature, there are examples showing that LbL film thickness depends on the roughness of the substrate. Trybała and coworkers [53] revealed that in the case of the formation of PAH/PSS multilayers on stainless steel, titanium and silicon plates with different roughness, the multilayers will be thicker if the substrate is rougher. The obtained results also indicate that the polyethyleneimine (PEI) anchoring layer has an influence on the film properties depending on the conditions of the film formation and on the roughness of the substrate. On surfaces with a higher roughness, a stronger effect of PEI is observed. However, in our case the differences in substrate roughness were too low for such an effect to be visible. Therefore, taking into account that the roughness of the two examined bare surfaces (Si and Ti, without adsorbed polyelectrolyte multilayers) is similar, and in order to explain the abovementioned trend, it is necessary to consider the interactions that occur between the substrate and the polyelectrolyte molecules. It is known from the literature that electrostatic interactions between substrates and polyelectrolyte molecules are the most dominant, but other interactions such as hydrogen bonds, hydrophobic and dipole–dipole interactions are not negligible [20]. Recently, fluorine-free superhydrophobic materials for multifunctional applications based on nano zinc oxide and polydimethylsiloxane (PDMS) were prepared using the spray-coating method [54]. Although in our study we concentrated on oxidized metal surfaces, it should be stated here that in recent years a series of papers that emphasize the importance of various alloys, especially in the field of applications of dual-ion batteries, has been published [55,56,57,58,59].

If we compare the isoelectric point values obtained for both investigated substrates, it could be concluded that the silicon surface is more negatively charged at examined conditions than the titanium surface. Therefore, it could be expected that the electrostatic interactions between PAH molecules and the surface will be more favorable than the interactions between titanium and PAH. Additionally, the effect of surface hydrophobicity should be taken into account and should not be underestimated.

## 4. Conclusions

In this paper, the influence of the type of metal substrates (silicon and titanium) on the PAH/PAA multilayer was investigated. We assumed that due to such a low surface roughness and the approximately equal thickness of the native oxide layers on the examined substrates, the properties of the PAH/PAA multilayer would depend primarily on the chemical nature of the surface and on the specific interactions with polyelectrolyte molecules. It was shown that the thickness of PAH/PAA multilayers containing 10 layers on the titanium surface is approximately three times higher than the thickness of the same film on the silicon surface. Also, the morphological properties of the obtained multilayers are different and these differences are reflected in the surface roughness of the samples, which can be related to the differences in obtained multilayer thickness. If it is assumed that the dominant interactions between the polyelectrolyte molecules and the substrate surface are electrostatic, it is possible to explain the observed results. As the isoelectric point differs for substrates and is higher for titanium oxide (4.1 ± 0.2) than for silica (3.5 ± 0.2), then this results in different properties of the multilayer. Thus, thinner and more compact films with a low surface roughness are expected in the case of strong interactions between the substrate surface and multilayers, while thicker and less compacted films with a higher surface roughness are expected in the case of weaker interactions. This was also supported by contact angle measurements that showed a higher surface free energy of the silicon substrate as compared to the titanium substrate. To conclude, based on the type of the substrate properties, the properties of the film can be predicted. Therefore, our study gives a further insight into the prediction of the behavior of polyelectrolyte multilayers. This in turn could enable the preparation of tuned PEMs, thus leading to improved applications of layer-by-layer structures.

## Figures and Tables

**Table 1:** Values of the oxide layer thickness (*d*) on silicon and titanium surface with the corresponding standard errors determined ellipsometrically as the average of the 10 measurements at various locations on the surface.

Metal Substrate	(*d* ± SE)/nm
Si	1.92 ± 0.02
Ti	6.70 ± 0.02

**Table 2:** Liquids used for tensiometric measurements and the values of the polar and dispersion components of the surface tension of liquids at 25 °C.

Liquid	$\sigma_{L}^{p}/\mathrm{mJ}\;m^{-2}$	$\sigma_{L}^{d}/\mathrm{mJ}\;m^{-2}$	$\sigma_{L}/\mathrm{mJ}\;m^{-2}$	References
Deionised water	51.0	21.8	72.8	[45]
Glycerol	26.4	37.0	63.4	[46]
Toluene	2.1	25.7	27.8	[47]
Formamide	19.0	39.0	58.0	[48]
*N*,*N*-Dimethylformamide	8.1	29.0	37.1	[47]
Dimethyl sulfoxide	8.0	36.0	44.0	[48]

**Table 3:** Thickness of PAH/PAA multilayers on silicon and titanium substrates determined by ellipsometry and atomic force microscopy.

Metal Substrate	*d*_elips/_nm	*d*_AFM_/nm
Si	10.1 ± 0.1	9.42 ± 0.1
Ti	30.5 ± 1.0	27.8 ± 1.0

## Data Availability

Not applicable.
